# Supplementary material for: Association between oral microbiome and seven types of cancers in East Asian population: a two-sample Mendelian randomization analysis
Source: Front Mol Biosci. 2023 Nov 21;10:1327893. doi: 10.3389/fmolb.2023.1327893 (PMC10702768; doi:10.3389/fmolb.2023.1327893)
Supplement: Supplementary file 1 [file DataSheet1.ZIP › Supplementary Materials/MR plots of saliva/saliva═╝/Gastric cancer/pheno.499_to_gastric cancer_leave_one_out.pdf]

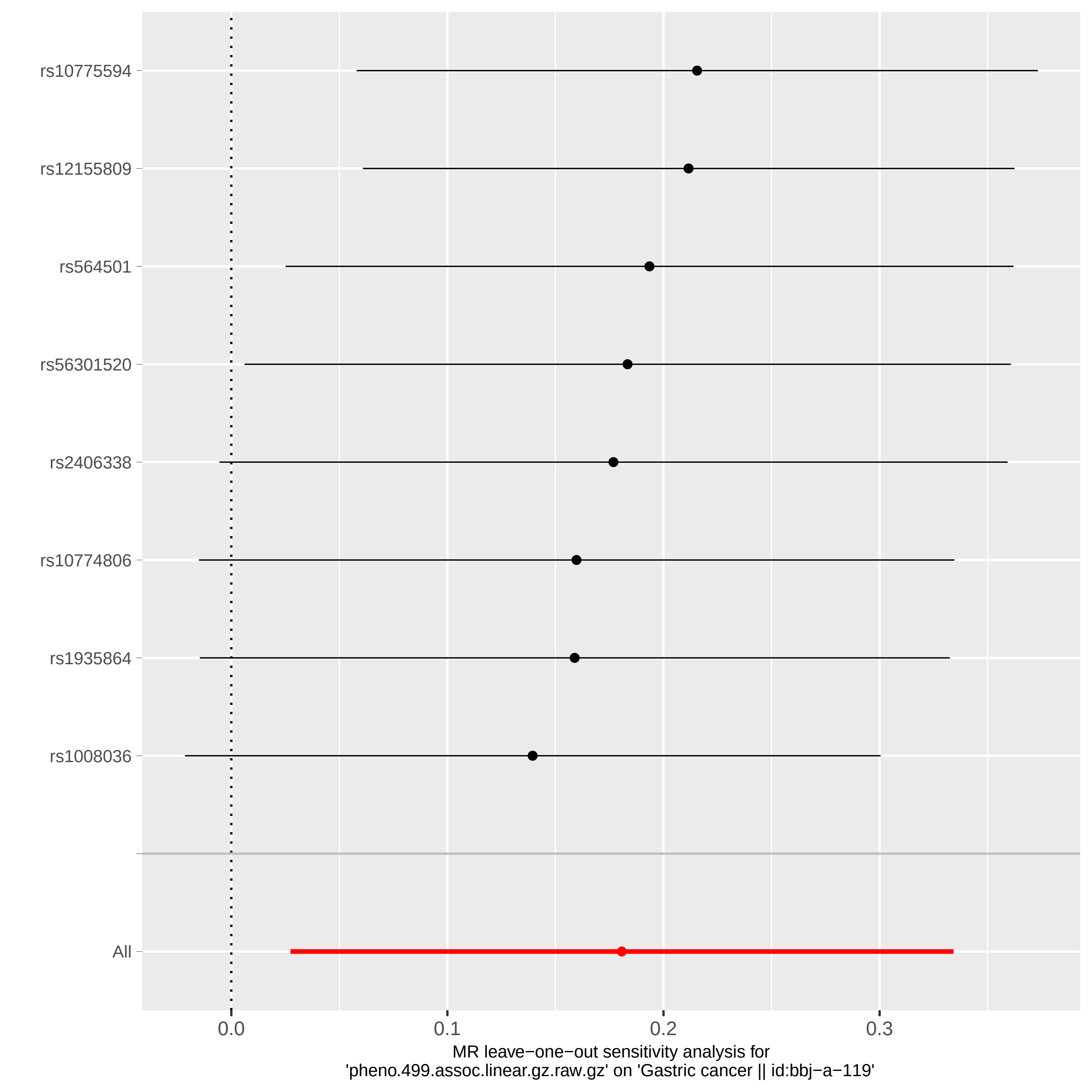
rs10775594
rs12155809
rs564501
rs56301520
rs2406338
rs10774806
rs1935864
rs1008036
All
0.0
0.1
0.2
0.3
MR leave−one−out sensitivity analysis for
'pheno.499.assoc.linear.gz.raw.gz' on 'Gastric cancer || id:bbj−a−119'
